# Novel Essential Amino Acid Supplements Following Resistance Exercise Induce Aminoacidemia and Enhance Anabolic Signaling Irrespective of Age: A Proof-of-Concept Trial

**DOI:** 10.3390/nu12072067

**Published:** 2020-07-12

**Authors:** Matthew J. Lees, Oliver J. Wilson, Erin K. Webb, Daniel A. Traylor, Todd Prior, Antonis Elia, Paul S. Harlow, Alistair D. Black, Paul J. Parker, Nick Harris, Michael Cooke, Christopher Balchin, Mathew Butterworth, Stuart M. Phillips, Theocharis Ispoglou

**Affiliations:** 1 Department of Physical Education and Sport Sciences, Faculty of Education and Health Sciences, University of Limerick, V94 T9PX Limerick, Ireland; 2 Carnegie School of Sport, Leeds Beckett University, Leeds LS6 3QS, UK; o.j.wilson@leedsbeckett.ac.uk (O.J.W.); alistair.black@leedsbeckett.ac.uk (A.D.B.); paul.parker@uhb.nhs.uk (P.J.P.); nickjohnharris@btinternet.com (N.H.); michael.cooke2@bthft.nhs.uk (M.C.); c.balchin@leedsbeckett.ac.uk (C.B.); m.butterworth9342@student.leedsbeckett.ac.uk (M.B.); t.ispoglou@leedsbeckett.ac.uk (T.I.); 3 Exercise Metabolism Research Group, Department of Kinesiology, McMaster University, Hamilton, ON L8S 4L8, Canada; webbe1@mcmaster.ca (E.K.W.); traylord@mcmaster.ca (D.A.T.); priort@mcmaster.ca (T.P.); phillis@mcmaster.ca (S.M.P.); 4 Division of Environmental Physiology, School of Chemistry, Bioengineering and Health, KTH Royal Institute of Technology, 171 65 Stockholm, Sweden; antonise@kth.se; 5 School of Life Sciences, University of Nottingham, University Park, Nottingham NG7 2RD, UK; paul.harlow@nottingham.ac.uk

**Keywords:** leucine, muscle mass, sarcopenia, anabolism, mTORC1, aging

## Abstract

We investigated the effects of ingesting a leucine-enriched essential amino acid (EAA) gel alone or combined with resistance exercise (RE) versus RE alone (control) on plasma aminoacidemia and intramyocellular anabolic signaling in healthy younger (28 ± 4 years) and older (71 ± 3 years) adults. Blood samples were obtained throughout the three trials, while muscle biopsies were collected in the postabsorptive state and 2 h following RE, following the consumption of two 50 mL EAA gels (40% leucine, 15 g total EAA), and following RE with EAA (combination (COM)). Protein content and the phosphorylation status of key anabolic signaling proteins were determined via immunoblotting. Irrespective of age, during EAA and COM peak leucinemia (younger: 454 ± 32 µM and 537 ± 111 µM; older: 417 ± 99 µM and 553 ± 136 µM) occurred ~60–120 min post-ingestion (younger: 66 ± 6 min and 120 ± 60 min; older: 90 ± 13 min and 78 ± 12 min). In the pooled sample, the area under the curve for plasma leucine and the sum of branched-chain amino acids was significantly greater in EAA and COM compared with RE. For intramyocellular signaling, significant main effects were found for condition (mTOR (Ser2481), rpS6 (Ser235/236)) and age (S6K1 (Thr421/Ser424), 4E-BP1 (Thr37/46)) in age group analyses. The phosphorylation of rpS6 was of similar magnitude (~8-fold) in pooled and age group data 2 h following COM. Our findings suggest that a gel-based, leucine-enriched EAA supplement is associated with aminoacidemia and a muscle anabolic signaling response, thus representing an effective means of stimulating muscle protein anabolism in younger and older adults following EAA and COM.

## 1. Introduction

Dietary protein intake and resistance exercise (RE) are recognized as important strategies for the stimulation of muscle protein synthesis (MPS) and the promotion of hypertrophy and retention of muscle mass. Amino acid availability is a key determinant of muscle protein synthesis (MPS; [1]). The consumption of different proteins can affect the amplitude and duration of MPS in the postprandial state [2] and may yield varying patterns of aminoacidemia [3,4]. The amino acid profile of the protein consumed, its digestion and absorption kinetics, and the time course of ingestion (i.e., bolus or constant intake) may all influence the postprandial MPS response [2,4,5,6]. The essential amino acids (EAAs) are particularly important, given their primary role in the amino acid-induced stimulation of MPS [7]. Among the EAAs, leucine has been described as a pharmaconutrient for the prevention and treatment of sarcopenia [8], possessing a distinct ability to stimulate skeletal muscle anabolism [9,10,11]. Leucine acts as a key anabolic signal for mRNA translation via the mammalian target of rapamycin complex 1 (mTORC1) pathway, in particular eukaryotic translation initiation factor 4E-binding protein 1 (4E-BP1), ribosomal protein S6 kinase (S6K1) and ribosomal protein S6 (rpS6) [10,12]. Furthermore, leucine appears to be required for maximal S6K1 activity in younger persons following resistance exercise.

Emerging work has highlighted the role of amino acid sensors, such as the Sestrins, in the mTORC1 pathway [13]. The Sestrins are a highly conserved family of multifunctional, stress-inducible proteins that have gained attention for their roles in metabolic homeostasis, disease, aging, and as key mediators of exercise benefits [14,15,16]. Nevertheless, to date, limited studies have explored the impact of aging on skeletal muscle Sestrin2 protein content in conjunction with dietary protein provision and RE.

When coupled with protein supplementation strategies, RE has a potentiating effect on translational regulation and MPS through mTORC1 signaling [17]. In isolation, blunted anabolic signaling responses have been observed in older adults following acute [18,19] and chronic RE [20,21], as well as the provision of dietary or intravenous EAAs [22,23]. There is also evidence that muscle protein anabolism is blunted or delayed in older adults when protein and RE are combined [24,25]. This phenomenon has been described as ‘anabolic resistance’ [11,26], although several studies have disputed its existence [27,28,29]. It appears that the optimisation of protein nutrition, and importantly leucine content, in conjunction with RE above a specific volume/intensity threshold is paramount for attaining ‘youthful’ anabolic responses in older adults [30].

Age-associated sarcopenia is characterized by a decline in skeletal muscle strength, quantity or quality, and physical performance [31]. It poses numerous consequences for older people, such as functional decline, an increased risk of falls, physical disability, and early mortality [32,33]. Dietary protein and RE are crucial for the management and prevention of sarcopenia, offering substantial therapeutic potential when applied in synergy [30,34]. Worryingly, a recent study of non-institutionalised older adults over 51 years of age (*n* = 11,680) reported that 31–50% did not even meet the recommended daily allowance (RDA) for protein intake (0.8 g/kg^−1^/body mass^−1^/day^−1^) [35]. This is a concern, as several authors have advised intakes exceeding 1.0 g/kg^−1^/body mass^−1^/day^−1^ of high-quality protein [36,37,38]. Although older persons may also be less responsive to the anabolic stimulus of low dose amino acid intake, this can potentially be overcome with higher amounts of protein or EAAs, thus supporting the rationale for increased protein intake in this population [36,39,40]. The fact that a large proportion of older adults fail to meet the present RDA for dietary protein, let alone the advised optimal intakes for health outcomes, suggests a need for efficient and effective supplementation methods. This is a challenge, considering that dietary and whey proteins have a well-established, dose-dependent satiating effect [41,42] that poses challenges for individuals with compromised appetite and/or dysregulated appetite hormones [43,44]. Recent work in our laboratory using a novel gel-based, leucine-enriched (40%) EAA formulation found no detrimental effects on subjective appetite responses, plasma peptide tyrosine tyrosine (PYY), or subsequent *ad libitum* energy intake in older women [45,46].

The purpose of this proof-of-concept study was to investigate the effects of a novel, leucine-enriched EAA gel alone and in combination with RE on aminoacidemia and intracellular mTORC1 signaling in healthy younger and older volunteers. We hypothesized that ingestion of EAA alone or in combination with RE would result in a more favourable aminoacidemic response that may support anabolic signaling. In turn, we hypothesized that ingestion of EAA following a bout of RE would result in augmented skeletal muscle anabolic signaling responses when compared to EAA or RE alone.

## 2. Materials and Methods

### 2.1. Participants

Seven (*n* = 7) younger (18–45 years; 4 males, 3 females) and seven (*n* = 7) older (60–80 years; 4 males, 3 females) volunteers took part in the research study. Participants were recruited based on their completion of a prior study [47]. All participants were independently living and free from cardiovascular, endocrine, or other metabolic disease. Participants were excluded if they smoked, used estrogens within the previous three months, had a pacemaker, or were taking any medication known to affect protein metabolism (i.e., anabolic steroids, corticosteroids, and/or peripheral vasodilators). Younger females were excluded if they were taking the oral contraceptive pill. Baseline characteristics are provided in Table 1. Informal verbal and written informed consent were provided by all participants. The study was granted ethical approval by the Yorkshire and the Humber–Bradford Leeds Research Ethics Committee (REC No: 17/YH/0238) and all procedures were conducted in accordance with the Helsinki Declaration of 1964.

### 2.2. Study Design

Participants reported to the laboratory on five separate occasions. The first visit consisted of preliminary screening and tests of anthropometry, functional performance, body composition by dual-energy X-ray absorptiometry (DXA), and familiarization with the lower body strength testing protocol. Diet diaries were provided and participants were instructed to record all food and fluid consumed for 72 h. The second visit verified the one-repetition maximum (1RM) for the leg extension test and enabled the return of diet diaries for analysis. Briefly, the subsequent three experimental trials consisted of the following conditions: essential amino acids (EAAs), resistance exercise (RE), or both in combination (COM). All laboratory visits were separated by a minimum of seven days and all trials were single-blind, randomized, and counterbalanced. Muscle biopsies were performed at baseline in the first experimental trial, and 2 h post-condition in all trials, to enable the assessment of intramuscular anabolic signaling proteins.

### 2.3. Experimental Procedures

Participants were screened and baseline measures were collected in congruence with the procedures outlined previously [47]. Diet diaries were obtained and analyzed using a commercially available software package (Nutritics v5.02, Nutritics Ltd., Dublin, Republic of Ireland) to establish habitual food and fluid intake. At the second laboratory visit, diaries were returned to participants with bespoke instructions to attain energy balance and consume a moderate protein intake of 1.0 g/kg^−1^/day^−1^ [48]. These intakes were replicated for 72 h prior to all experimental trials.

On the morning of each experimental trial, participants arrived at 08.00 h following an overnight fast and avoidance of strenuous exercise for 48 h prior to arrival at the laboratory [19]. A 22-gauge Teflon catheter was inserted into a cephalic or median basilic vein by a trained phlebotomist. Blood samples were obtained at baseline (−60 min), immediately following each condition (0 min), and at 30, 60, 90, and 120 min. Due to difficulties with cannulation, data are reported for five participants per age group.

In the first experimental trial, immediately following cannula insertion a percutaneous skeletal muscle biopsy was obtained from the *m. vastus lateralis* under local anaesthesia (0.5% bupivacaine hydrochloride; Marcaine^®^, AstraZeneca Ltd., Cambridge, UK) using a UCH (University College Hospital) biopsy needle and manual suction [49]. Samples were blotted, freed of excess connective tissue and fat, then placed in an Eppendorf tube and rapidly frozen in liquid nitrogen prior to storage at −80 °C for subsequent analysis.

In RE and COM, participants completed a bout of unilateral leg extension exercise on a commercially available machine (Cybex VRS Prestige, Cybex International, Medway, MA, USA) immediately following baseline measures. This was initiated with a dynamic warmup, emphasizing the lower body musculature, for 5 min followed by 10 repetitions at 40% 1RM. Following this, participants completed 8 sets of 10 repetitions at 70% 1RM, with 3 min rest between sets and a total bout duration of ~40 min [19,24]. Measures were taken to ensure that the exercised leg always received the biopsy, with the biopsied leg selected at random and alternated between trials.

In EAA and COM, participants consumed two carbohydrate-free and fat-free, EAA-enriched gels (50 mL each; 30 kcals/gel) 60 min following basal measures. These gels were adapted as carbohydrate-free and artificially-sweetened variants of a formulation previously used in our laboratory to investigate appetite responses in older adults [45,46]. The EAA composition of each gel is provided in Table 2 and was derived from earlier work by Ispoglou et al. [50]. All EAAs were purchased from Fagron UK Ltd. (Newcastle upon Tyne, UK) and developed into final formulations with the assistance of a product developer to the food industry based at Askham Bryan College (York, UK). Ingestion of the two gels provided 15 g of EAAs, the equivalent amount obtained from approximately 30 g of quality protein, and delivered a high amount (~6 g, or 40%) of leucine [9,36,45,51]. Gels were accompanied with 175 mL water, which was provided at the same time in RE to maintain consistency across all three trials.

Following gel consumption, participants observed 2 h of seated rest [52,53], with blood samples collected every 30 min, before a second and final muscle biopsy was obtained 120 min after the cessation of each condition, via a separate proximal incision [53,54].

### 2.4. Immunoblot Analysis

**Protein extraction.** Briefly, muscle tissue samples (~20 mg) were homogenised in lysis buffer (25 mM Tris pH 7.2; 1 × phosphatase inhibitor tablet (PhosSTOP^TM^, Roche, Mississauga, ON, Canada) per 10 mL of buffer; 1 × protease inhibitor cocktail (cOmplete^TM^, Roche, Mississauga, ON, Canada) per 10 mL of buffer; 0.5% Triton X-100) in a bead homogeniser for 40 s at 20 Hz (TissueLyser II, Qiagen, Hilden, Germany). Tissue homogenates were centrifuged at 10,000 *g* for 10 min at 4 °C and the supernatant removed for storage at −80 °C prior to analysis. The total protein concentration was determined using a Pierce^TM^ bicinchoninic acid (BCA) protein assay kit (Thermo Fisher Scientific, Waltham, MA, USA). 

**SDS-PAGE and Western blotting.** The extent of intracellular signaling protein content and phosphorylation was assessed using sodium dodecyl sulfate polyacrylamide gel electrophoresis (SDS-PAGE) and Western blotting. Working samples (200 µL) of equal concentration (0.5 µg/µL) were prepared by diluting muscle homogenates in Laemmli buffer (Bio-Rad, Hercules, CA, USA), *β*-mercaptoethanol (M6250, Sigma Aldrich, Toronto, ON, Canada) and deionised water, followed by heating at 95 °C for 5 min. Subsequently, equal amounts of protein (7.5 µg) were loaded onto 4–15% Criterion^TM^ TGX Stain-Free^TM^ protein gels (Bio-Rad) and electrophoresed at 200V for 45 min. All gels were run with a protein ladder (Precision Plus Protein^TM^ All Blue Protein Standard, Bio-Rad) and calibration curve of pooled muscle homogenates (i.e., 7.5, 10, 12.5, 15 µL). Proteins were then wet-transferred to nitrocellulose membranes using the Trans-Blot Turbo^TM^ Transfer System (Bio-Rad). Gels and membranes were visually inspected pre- and post-transfer using a ChemiDoc^TM^ MP Imaging System (Bio-Rad).

Following transfer, membranes were blocked for 1 h in 5% bovine serum albumin (BSA; Bioshop, Canada) and probed with primary antibodies overnight (see Antibodies below for concentrations) for 14 h at 4 °C, under gentle agitation. The next morning, blots were washed in tris-buffered saline with Tween 20 (TBST) for 3 × 5 min and then incubated in secondary antibody for 1 h 10 min at room temperature. The washing step was then repeated, and membranes immersed in detection reagent (Clarity Max^TM^ Western ECL Blotting Substrate, Bio-Rad, Hercules, CA, USA) for 5 min immediately prior to the capture of chemiluminescent signals (ChemiDoc^TM^ MP Imaging System, Bio-Rad, Hercules, CA, USA). 

Densitometric analysis of protein bands was performed using bespoke software (Image Lab, Version 6.0.1, Bio-Rad). Total and phosphorylated protein density values were normalized to total protein using the calibration curve obtained from each gel [55]. Phosphoproteins were expressed relative to their corresponding total proteins and quantified as fold change from baseline [56] unless otherwise stated.

**Antibodies.** Primary antibodies were purchased from Cell Signaling Technology (Danvers, MA, USA) unless otherwise stated. Antibodies targeting total protein content were as follows: Akt (1:1000; #4691), mTOR (1:1000; #2972), focal adhesion kinase (FAK; 1:1000; #3285), 4E-BP1 (1:1000; #9644), S6K1 (1:1000; #9202), rpS6 (1:1000; #2217), and Sestrin2 (1:1000; Abcam, #ab178518). The following phospho-specific antibodies were used: phospho-Akt (Ser473; 1:1000; #9271), phospho-mTOR (Ser2481; 1:1000; #2974), phospho-FAK (Tyr397; 1:1000; #8556), phospho-4E-BP1 (Thr37/46; 1:1000; #2855), phospho-S6K1 (Thr 421/Ser424; 1:1000; #9204), and phospho-rpS6 (Ser235/236; 1:1000; #2211). Anti-rabbit IgG horseradish peroxidase-conjugated secondary antibody was purchased from Cell Signaling Technology (1:3000; #7074).

**Plasma variables.** Venous blood was collected in ethylenediaminetetraacetic acid (EDTA) tubes (Vacutainer^®^, Becton Dickinson, Franklin Lakes, NJ, USA), centrifuged at 1000 *g* for 10 min at 4 °C, and plasma was aliquoted and frozen at −80 °C for subsequent analysis. 

Plasma branched-chain amino acid (BCAA) concentrations were assessed using the EZ:faast^TM^ amino acid analysis kit for gas chromatography-mass spectrometry (GC-MS; Phenomenex, Torrance, CA, USA) in line with previous work [4]. Samples were prepared and derivatized as indicated using the protocol provided by Phenomenex. Briefly, samples were analyzed using an Agilent 7890A GC System with 5975C MSD using electron impact ionization and a 7693 Autosampler (Agilent Technologies, Santa Clara, CA, USA) with the following mass spectrometer settings: source 240 °C; quad 180 °C; auxiliary 310 °C. Samples were separated using the stock Phenom cgo-7169ZB-AAA (325 °C; 10 m × 250 µm × 0.25 µm) GC column provided with the kit. Aliquots of 2 µL of the derivatized sample or standard were delivered by autosampler and set to run a 15:1 split mode injection at 250 °C. The GC was operated in constant pressure mode at approximately 2.9 psi producing a starting flow rate of 1.4 mL/min with an initial oven temperature of 110 °C. The temperature ramp was as follows: 110 °C with no hold, followed by a ramp of 30 °C/min to 320 °C, followed by 1 min hold and 1 min post-run at 320 °C for a total run time of 8 min. Chromatographic data were originally collected using scan mode (50–450 m/z) to confirm peak identity, target ions and retention times using the Agilent library file provided by Phenomenex. Subsequently, based on this data a Selected Ion Monitoring (SIM) method was employed, using a series of six ion groups representing the desired target ions necessary to quantify the sample AAs. These ions were confirmed to be the same ions suggested by the manufacturer. Chromatographs were evaluated and quantified using the ChemStation enhanced data analysis software (Agilent Technologies, Santa Clara, CA, USA) alongside the methodological instructions and electron ionization spectra database provided with the kit. The method employed three levels of AA standard mixtures in conjunction with an internal standard (norvaline) to calculate the response factors used for quantitation. These response factors were used to generate standard curves for leucine, isoleucine, and valine. Linear regression of these curves provided the coefficients necessary to determine plasma BCAA concentrations.

Plasma glucose concentrations were analyzed using hexokinase/glucose-6-phosphate dehydrogenase methodology and plasma insulin concentrations were determined by chemiluminescent microparticle immunoassay as previously described [4]. The homeostatic model assessment of insulin resistance (HOMA-IR) was determined by the formula: fasting insulin (µIU/L) x fasting glucose (nmol/L)/22.5 [57].

### 2.5. Statistical Analysis

All statistical analyses were conducted using bespoke packages (i.e., effsize, ezANOVA, MOTE, psych, survival) in the R programming language. Figures were generated using GraphPad Prism (Version 8, GraphPad Software, La Jolla, CA, USA).

Variables were checked for multicollinearity, homogeneity of variance, outliers, and normality prior to performing statistical tests. For instances in which data were not normally distributed, these variables were log-transformed. Baseline characteristics were compared between age groups using independent *t*-tests. For immunoblot and plasma time series data, two-way repeated measures analyses of variance (ANOVA) were performed on pooled data to explore main effects of time, condition (i.e., EAA, RE, COM), and time x condition interaction effects. Peak concentration (C_max_) and time to peak concentration (T_max_) were determined and area under the curve (AUC) was calculated using the trapezoidal method. One-way repeated measures ANOVA was performed to explore differences in C_max_, T_max_ and AUC in pooled plasma data. For age group comparisons, mixed model ANOVA was conducted, with one within-subject effect (i.e., condition) and one between-group effect (i.e., age). For all ANOVA tests, significant main and interaction effects were explored using appropriate post-hoc tests with Holm–Bonferroni adjustment for multiple comparisons. 

Where appropriate, effect sizes (Cohen’s *d*) were calculated and interpreted as follows: trivial (<0.2), small (0.2–0.5), moderate (0.5–0.8), and large (≥0.8) [58]. For all analyses, alpha was accepted as *p* ≤ 0.05. Based on a large effect size threshold (*d* = 0.8), for pooled analyses a total sample size of 12 was deemed necessary to obtain a statistical power of at least 80% with the selected alpha. Effects equal to or greater than *d* = 0.9 required a total sample size of nine to obtain equivalent power. Sample size calculations were performed using G*Power [59]. Data are presented as means ± SEM (standard error of the mean) unless otherwise stated. 

## 3. Results 

### 3.1. Baseline Characteristics

The older group had significantly lower dominant leg 1RM strength and lower body MQ than the younger group (Table 1). No other significant differences in physical measures, body composition, or functional performance were found (all *P* > 0.05).

### 3.2. Plasma Leucine, Branched-Chain Amino Acids, Insulin, and Glucose

Fasting and peak concentrations, time to peak concentration, and AUC values for all plasma variables in each age group are shown in Table 3. 

**Figure 1:**
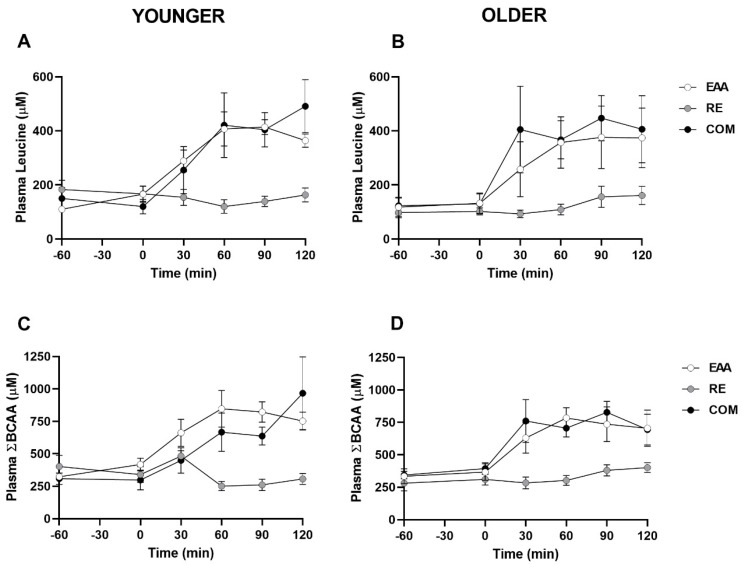
Plasma leucine and branched-chain amino acid concentrations in younger (**A**,**C**) and older (**B**,**D**) adults following EAA, RE and COM (*n* = 5 per group). Data are means ± SEM.

**Leucine.** For time series data in the pooled sample, analysis showed that there were significant main effects of time, condition, and a time x condition interaction (all *p* < 0.01). Plasma leucine tended to be higher 90 min after ingestion of EAA compared to baseline (382 ± 54 vs. 114 ± 17 µM; *p* = 0.09, *d* = 2.11). In RE, plasma leucine did not significantly change from baseline (all *p* > 0.99). In COM, plasma leucine tended to be elevated at 60 min post-ingestion, compared with baseline (139 ± 25 vs. 394 ± 66 µM; *p* = 0.07, *d* = 1.62), and was significantly greater at 90 min (426 ± 50 µM; *p* < 0.01, *d* = 2.30) and 120 min (449 ± 76 µM; *p* = 0.04, *d* = 1.74), compared with baseline. When data were split by age group, a significant main effect was observed for condition (*p* < 0.001), but not age (*p* = 0.88) or age x condition interaction (*p* = 0.96). In the younger group, plasma leucine tended to be higher 90 min following EAA compared with baseline (*p* = 0.08, *d* = 3.81; Figure 1A), whereas all other comparisons were non-significant (*p* > 0.23). In the older group, decreased plasma leucine was observed 30 min after the conclusion of RE (*p* = 0.02, *d* = 0.29; Figure 1B), with no other significant differences observed (*p* > 0.56).

Peak postprandial leucine concentrations (C_max_) in the pooled sample differed significantly (*p* < 0.01) with EAA (436 ± 49 µM; *p* < 0.01, *d* = 2.23) and COM (545 ± 83 µM; *p* < 0.01, *d* = 1.97) greater than RE (167 ± 22 µM). EAA and COM did not significantly differ (*p* = 0.27, *d* = 0.51). After splitting for age, leucine C_max_ was significantly different between conditions (*p* < 0.001; Table 3). In the young, EAA and COM were similar (*p* = 0.52, *d* = 0.45), and higher than RE (*p* = 0.02, *d* = 4.30; *p* = 0.06, *d* = 2.03; respectively). In the older group, COM tended to be higher compared with RE (*p* = 0.06, *d* = 1.74) and was similar to EAA (*p* = 0.43, *d* = 0.51). EAA did not differ from RE despite a large effect (*p* = 0.13, *d* = 1.51).

Time to peak plasma leucine concentration (T_max_) in the pooled sample did not differ between conditions (*p* = 0.14). After splitting by age, T_max_ differed significantly (age x condition interaction: *p* = 0.01), occurring earlier in COM compared with RE in the older group (*p* = 0.01, *d* = 1.70). T_max_ did not differ significantly by condition in the younger group.

Leucine AUC in the pooled sample significantly differed between conditions (*p* < 0.01) with EAA (39272 ± 5299 µM; *p* < 0.01, *d* = 1.51) and COM (43094 ± 6612 µM; *p* < 0.01, *d* =1.29) greater than RE (16022 ± 1844 µM). EAA and COM did not significantly differ (*p* = 0.62, *d* = 0.20). After splitting by age, leucine AUC differed by condition (*p* = 0.002) but there were no main effects for age (*p* = 0.87) or age x condition interaction (*p* = 0.86). In the young, leucine AUC was greater in EAA compared with RE (*p* = 0.03, *d* = 3.82) and was similar to COM (*p* = 0.96, *d* = 0.02). In the older group, leucine AUC tended to be greater in COM compared with RE (*p* = 0.07, *d* = 1.73), with no other significant effects. 

**Branched-chain amino acids.** Time series data for plasma BCAA concentrations in the pooled sample showed that there were significant main effects of time, condition, and a time x condition interaction (all *p* < 0.01). In the EAA condition, plasma BCAA concentrations were significantly elevated at 60 min (815 ± 78 µM; *p* = 0.02, *d* = 2.63), 90 min (779 ± 74 µM; *p* = 0.01, *d* = 2.53), and 120 min (729 ± 74 µM; *p* = 0.03, *d* = 2.25) postprandially compared with baseline (329 ± 29 µM). In the RE condition, plasma BCAA did not significantly change during the trial (all *p* > 0.99). Following COM, plasma BCAA were elevated at 60 min (686 ± 77 µM; *p* = 0.03, *d* = 1.99) and 90 min (733 ± 60 µM; *p* < 0.001, *d* = 2.77) postprandially compared with baseline (327 ± 26 µM). Age group time series data are shown in Figure 1C and 1D, respectively. Concurrent with plasma leucine, BCAA concentrations were significantly different between conditions (*p* < 0.001) but there was no effect of age (*p* = 0.97) or age x condition interaction (*p* = 0.22). Although post-hoc testing was performed, no significant differences were found (all P > 0.24). 

Peak plasma BCAA concentrations in the pooled sample differed significantly (*p* < 0.01), with EAA (889 ± 56 µM; *p* < 0.01, *d* = 2.86) and COM (953 ± 142 µM; *p* < 0.01, *d* = 1.48) greater than RE (467 ± 35 µM). COM and EAA did not significantly differ (*p* = 0.66, *d* = 0.19). After splitting data for age, BCAA C_max_ was significantly different between conditions (*p* = 0.002), but there were no effects for age (*p* = 0.59) or age x condition interaction (*p* = 0.94). In the young, peak BCAA did not differ between groups (all *p* > 0.11; Table 3), whereas in the older group BCAA C_max_ was higher in EAA compared with RE (*p* = 0.01, *d* = 3.54) and tended to be higher in COM (*p* = 0.06, *d* = 2.16), with no difference between EAA and COM (*p* = 0.84, *d* = 0.14). 

For time to peak concentration, in pooled data a trend was found between conditions (*p* = 0.07). When data were split by age group, significant main effects for condition (*p* = 0.01) and age x condition interaction (*p* = 0.03) were found. However, post-hoc testing could not detect these differences (all *p* > 0.10). 

For BCAA AUC, a significant main effect was observed for condition (*p* < 0.01), with EAA (84023 ± 6838 µM; *p* < 0.01, *d* = 2.70) and COM (78347 ± 8221 µM; *p* < 0.01, *d* = 2.00) significantly greater than RE (39653 ± 2681 µM). EAA and COM did not significantly differ (*p* = 0.58, *d* = 0.24). After splitting by age group, a significant main effect was found for condition (*p* < 0.001), but not age (*p* = 0.78) or age x condition interaction (*p* = 0.53). AUC was higher in the younger group following EAA, compared with RE (*p* = 0.05, *d* = 2.80) and was not different to COM (*p* = 0.29, *d* = 0.62). In the older group, AUC was significantly higher after EAA, compared to RE (*p* = 0.04, *d* = 2.36) and tended to be higher in COM (*p* = 0.06, *d* = 2.50), with no difference between COM or EAA (*p* = 0.77, *d* = 0.20).

**Insulin.** For the pooled sample, significant main effects of time, condition, and time x condition interaction were found (*p* < 0.01). In EAA and RE, plasma insulin did not significantly differ from baseline values (9.1 ± 1.7 and 8.3 ± 1.8 µIU/mL, respectively; all *p* > 0.12). In COM, plasma insulin was significantly elevated at 0 min (17.8 ± 2.0 µIU/mL; *p* = 0.01; *d* = 2.79), at 30 min (14.2 ± 1.6 µIU/mL; *p* = 0.03, *d* = 2.11) and 60 min (12.7 ± 1.5 µIU/mL; *p* = 0.04, *d* = 1.74) postprandially, compared with baseline (6.7 ± 1.6 µIU/mL).

After splitting data by age group, significant main effects were found for age (*p* = 0.01) and condition (*p* < 0.001), but not age x condition interaction (*p* = 0.14). Post-hoc testing revealed no significant change in the younger group in EAA and RE, whereas in COM, insulin was significantly increased immediately post-condition (i.e., 0 min), compared to basal (*p* = 0.02, *d* = 3.51). In the older group, insulin was significantly elevated at 0 min (*p* = 0.01, *d* = 3.72) and higher at 30 min (*p* = 0.05, *d* = 1.91) compared with baseline in COM. Between groups, insulin was significantly greater in the older group at 60 min in EAA (*p* < 0.001, *d* = 4.57) and 0 min in COM (*p* = 0.01, *d* = 2.63). 

Peak insulin concentration differed between conditions in the pooled sample (*p* < 0.01). EAA was higher than COM (24.2 ± 2.0 vs. 18.6 ± 1.2 µIU/mL; *p* < 0.01, *d* = 1.07) and RE (14.7 ± 1.6 µIU/mL; *p* < 0.01, *d* = 1.64). COM was also significantly higher than RE (*p* < 0.01, *d* = 0.88). After splitting for age, significant main effects were found for age (*p* = 0.006) and condition (*p* < 0.001), but there was no age x condition interaction (*p* = 0.66; Table 3). In the young, peak insulin concentration tended to be higher in EAA and COM, compared with RE (both *p* = 0.07, *d* = 0.96 and *d* = 1.98, respectively) but did not differ between COM and EAA (*p* = 0.20). In the older group insulin C_max_ was higher in EAA compared with RE and COM (both *p* = 0.03; *d* = 2.29 and *d* = 2.01, respectively), and higher in COM compared with RE (*p* = 0.03, *d* = 0.71). In all conditions, insulin C_max_ was higher in the older group compared with the younger group (all *p* < 0.04; *d* = 1.59 - 2.10). 

No main effects were found for insulin T_max_ in the pooled sample (*p* = 0.87) or age groups (all *p* > 0.13). 

For insulin AUC, significant differences were found between conditions in the pooled sample (*p* < 0.01). EAA was greater than COM (1927.8 ± 161.8 vs. 1608.3 ± 124.2 µIU/mL; *p* = 0.03, *d* = 0.70) and RE (1188.5 ± 132.6 µIU/mL; *p* < 0.01, *d* = 1.58), respectively. COM was also greater than RE (*p* < 0.01, *d* = 1.03). After splitting groups by age, main effects were found for age (*p* = 0.03), condition (*p* < 0.001), and age x condition interaction (*p* = 0.02). In the younger group, there was a tendency for insulin AUC to be greater in EAA and COM, compared with RE (both *p* = 0.08; *d* = 2.60 and *d* = 2.40, respectively). In the older group, insulin AUC was greater in EAA (*p* = 0.003, *d* = 2.23) and COM (*p* = 0.003, *d* = 0.84), compared with RE and was greater in EAA compared with COM (*p* = 0.004, *d* = 1.38). Insulin AUC was significantly greater in the older group, compared with the younger group in EAA (*p* = 0.004, *d* = 3.06) but was not different for RE and COM (both *p* = 0.26, *d* = 0.96 and *d* = 1.07). 

**Glucose.** In pooled data, a significant main effect was found for time (*p* < 0.01) but not condition (*p* = 0.86) or time x condition interaction (*p* = 0.66). However, post-hoc testing revealed no significant changes in plasma glucose between any time point. When data were split by age group (Table 3), no significant main or interaction effects were found for plasma glucose in time series values, peak concentration, time to peak concentration, or AUC (all *p* > 0.20). 

### 3.3. Akt Signaling

**Figure 2:**
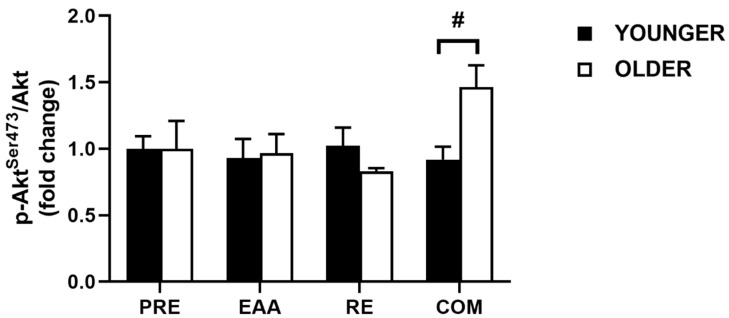
Phosphorylation of Akt at Ser473 2 h following EAA, RE, and COM in healthy younger (*n* = 7) and older (*n* = 7) individuals. The symbol (**#**) denotes statistical significance between age groups. Data are means ± SEM.

**Figure 3:**
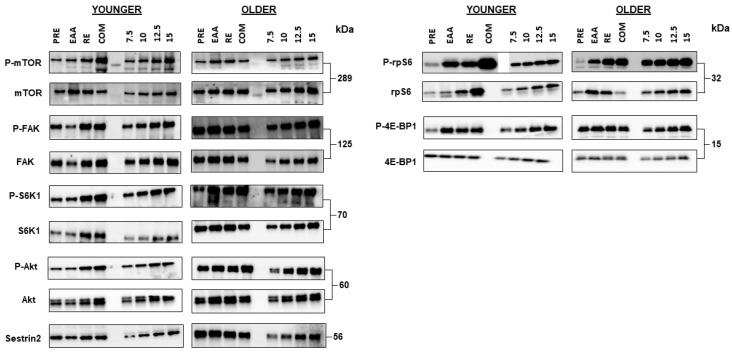
Representative blots for each total and phosphoprotein. Blots are in the following order: baseline, EAA, RE, and COM. The fifth lane is occupied by the protein ladder. To the right of the experimental blots, the loading controls (calibration curves) are shown (i.e., 7.5, 10, 12.5, and 15 µL of protein).

For pooled data, no main effect for time (*p* = 0.77), condition (*p* = 0.12), or time x condition interaction (*p* = 0.12) was found between EAA (0.95 ± 0.10; *d* = 0.13), RE (0.93 ± 0.07; *d* = 0.20), and COM (1.19 ± 0.12; *d* = 0.45) compared with PRE (1.00 ± 0.11). When data were split by age group, no significant main effect for age (*p* = 0.40) or condition (*p* = 0.17) was found for Akt*^Ser473^*phosphorylation; however, there was an age x condition interaction (*p* = 0.04; Figure 2; see Figure 3 for representative immunoblots). Akt*^Ser473^* phosphorylation was higher in the older group, compared with the younger group after COM, constituting a large effect (*p* = 0.05; *d* = 1.55). In the younger group, Akt*^Ser473^* phosphorylation did not differ significantly from baseline (all *p* > 0.80). 

### 3.4. mTORC1 Signaling

**Figure 4:**
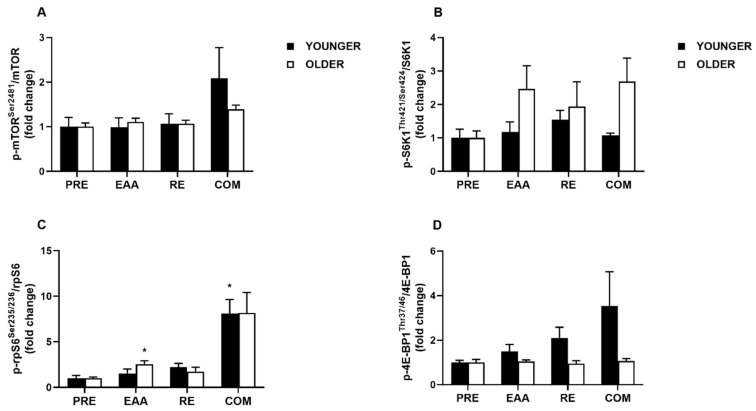
Phosphorylation of mTOR at Ser2481 (**A**), S6K1 at Thr421/Ser424 (**B**), rpS6 at Ser235/236 (**C**), and 4E-BP1 at Thr37/46 (**D**) following EAA, RE, and COM in healthy younger (*n* = 7) and older (*n* = 7) adults. The symbol (*) denotes statistical significance compared to baseline. Data are means ± SEM.

The phosphorylation activity of key mTORC1 pathway components in younger and older adults is shown in Figure 4. For mTOR*^Ser2481^* phosphorylation, there was a tendency towards a main effect of time (*p* = 0.08), a significant main effect for condition (*p* = 0.04), and an interaction effect (*p* = 0.04). However, post-hoc testing did not reveal any significant differences between EAA (1.05 ± 0.11; *d* = 0.13), RE (1.07 ± 0.11; *d* = 0.17), and COM (1.74 ± 0.35; *d* = 0.77) in relation to PRE (1.00 ± 0.11). When data were split by age group, significant main effects were found for condition (*p* = 0.02), but not age (*p* = 0.59), or age x condition interaction (*p* = 0.36; Figure 4A). Subsequent post-hoc testing did not detect any significant differences (all *p* > 0.25). In the younger group, a large increase following COM was observed (*d* = 0.81), but trivial effects after EAA (*d* = 0.01) and RE (*d* = 0.11), in comparison with baseline. The same pattern was found in the older group, with a large increase following COM (*d* = 1.65), but small following EAA (*d* = 0.49) and RE (*d* = 0.30).

For SK61*^Thr421/Ser424^* phosphorylation, in the pooled sample there was a main effect of time (*p* = 0.01), but not condition (*p* = 0.98) or time x condition interaction (*p* = 0.98). However, post-hoc testing did not reveal any significant differences (all *p* > 0.17) between EAA (1.82 ± 0.41; *d* = 0.71), RE (1.74 ± 0.38; *d* = 0.68), and COM (1.88 ± 0.40; *d* = 0.77) in relation to PRE (1.00 ± 0.16). When data were split by age group, a main effect was found for age (*p* = 0.01), but not condition or age x condition interaction (both *p* > 0.28; Figure 4B). Post-hoc testing did not detect any significant differences (all *p* > 0.16). However, a large effect was found between age groups after COM (*d* = 1.23) and EAA (*d* = 0.90), but not RE (*d* = 0.27). Within groups, a moderate effect was found after RE in the younger group, compared with baseline (*d* = 0.77), with small and trivial effects after EAA (*d* = 0.23) and COM (*d* = 0.15), respectively. Comparatively, in the older group large effects were found following COM (*d* = 1.24) and EAA (*d* = 1.07), with moderate after RE (*d* = 0.65).

For rpS6*^Ser235/236^* phosphorylation, in the pooled sample there were significant main effects of time, condition, and time x condition interaction (all *p* < 0.001). Compared with PRE (1.00 ± 0.17), rpS6*^Ser235/236^* phosphorylation was greater after EAA (2.04 ± 0.32; *p* = 0.05; *d* = 1.08), RE (1.99 ± 0.31; *p* = 0.05; *d* = 1.06), and COM (8.13 ± 1.32; *p* < 0.001; *d* = 2.03). COM was also greater compared to RE (*p* < 0.01; *d* = 1.72) and EAA (*p* < 0.01; *d* = 1.70). When data were split by age group, a main effect was found for condition (*p* < 0.001), but not age or age x condition interaction (both *p* > 0.73; Figure 4C). Post-hoc testing revealed no significant differences between age groups (all *p* > 0.52), but a large effect was found between groups following EAA (*d* = 0.87), moderate after RE (*d* = 0.44), and trivial after COM (*d* = 0.02). In the younger group, a large and significant effect was found after COM compared with baseline (*p* = 0.05; *d* = 2.37), with no other statistically significant effects (*p* > 0.30). Large and moderate effects were noted following RE (*d* = 1.30) and EAA (*d* = 0.50), respectively. In the older group, a large and significant effect was found following EAA (*p* = 0.05; *d* = 1.99), with no other statistically significant differences (*p* > 0.10). However, large effects were observed following COM (*d* = 1.70) and RE (*d* = 0.79), respectively.

For 4E-BP1*^Thr37/46^* phosphorylation, in the pooled sample there was a significant main effect of time (*p* = 0.05), but not condition (*p* = 0.52) or time x condition interaction (*p* = 0.52). Post-hoc testing revealed no significant differences (all *p* > 0.43) between EAA (1.25 ± 0.16; *d* = 0.52), RE (1.50 ± 0.28; *d* = 0.64), and COM (2.26 ± 0.81; *d* = 0.58) in relation to PRE (1.00 ± 0.08). When data were split by age group, a main effect for age was found (*p* = 0.02), but not condition or age x condition interaction (both *p* > 0.20; Figure 4D). Subsequent post-hoc testing did not detect any significant differences (all *p* > 0.19). However, large effects were found between age groups following RE (*d* = 1.24) and COM (*d* = 0.85), with moderate after EAA (*d* = 0.76). In the younger group, large effects were found for 4E-BP1*^Thr37/46^* phosphorylation after RE (*d* = 1.21), COM (*d* = 0.88) and EAA (*d* = 0.82). By comparison, in the older group only trivial effects were found for each condition (*d* = 0.13 – 0.18).

### 3.5. FAK Signaling

**Figure 5:**
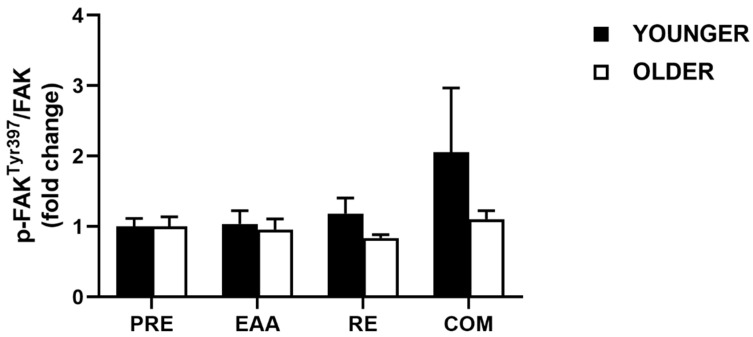
Phosphorylation of FAK at Tyr397 following EAA, RE and COM in healthy young (*n* = 7) and older (*n* = 7) individuals. Data are means ± SEM.

For pooled data, no significant main effect of time (*p* = 0.66), condition (*p* = 0.10), or time x condition interaction (*p* = 0.10) was found in FAK*^Tyr397^* phosphorylation between EAA (1.00 ± 0.11; *d* = 0.00), RE (1.01 ± 0.12; *d* = 0.03), and COM (1.58 ± 0.46; *d* = 0.47) compared with PRE (1.00 ± 0.06). When data were split by age group, no main effects were found for age, condition, or age x condition interaction (all *p* > 0.22; Figure 5). A large effect was found between age groups following RE (*d* = 0.82), moderate after COM (*d* = 0.56), and trivial after EAA (*d* = 0.18). In the young, a moderate difference was found between baseline FAK*^Tyr397^* phosphorylation and COM (*d* = 0.62), small after RE (*d* = 0.40) and trivial after EAA (*d* = 0.09). Conversely, in the older group, a moderate difference was found after RE (*d* = 0.59), small following COM (*d* = 0.31) and trivial after EAA (*d* = 0.11).

### 3.6. Sestrin2 Protein Content

**Figure 6:**
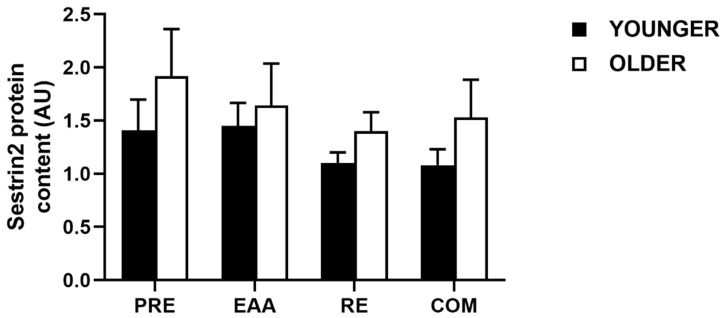
Sestrin2 protein content (arbitrary units; AU) at baseline and 2 h following EAA, RE, and COM in healthy younger (*n* = 7) and older (*n* = 7) individuals. Data are means ± SEM.

For total Sestrin2 protein content, no significant main effect of time (*p* = 0.26), condition (*p* = 0.36), or time x condition interaction (*p* = 0.36) was found between PRE (1.67 ± 0.26 arbitrary units (AU)), EAA (1.55 ± 0.22 AU), RE (1.26 ± 0.11 AU), and COM (1.31 ± 0.20 AU) when data were pooled (*d* = 0.08–0.54). Similarly, when data were split by age group, no significant main effects were observed for age (*p* = 0.25), condition (*p* = 0.24) or age x condition interaction (*p* = 0.90; Figure 6). At baseline, Sestrin2 protein content was moderately greater in the older group (*d* = 0.51), with further moderate effects in RE (*d* = 0.78) and COM (*d* = 0.63), but a small effect in EAA (*d* = 0.22). 

In the young, a trivial difference was found between baseline and post-EAA Sestrin2 protein content (*d* = 0.06), whereas moderate decreases were noted after RE (*d* = 0.54) and COM (*d* = 0.54). In the older group, a small decrease was observed post-EAA (*d* = 0.25), a moderate decrease after RE (*d* = 0.58) and a small decrease after COM (*d* = 0.37).

## 4. Discussion

Our aim in this pilot study was to provide evidence concerning the efficacy of a novel gel-based, leucine-enriched EAA supplement on plasma aminoacidemia and skeletal muscle anabolic signaling in healthy younger and older adults. In the EAA and COM trials, we observed robust leucinemia in pooled and age group data that followed a comparable kinetic profile. When the gels were ingested following RE, there was stimulation of markers of anabolic signaling in the skeletal muscle of younger and older individuals, an effect that was enhanced when compared with EAA or RE alone. These data support the synergistic influence of dietary EAAs when consumed post-RE on plasma amino acids and markers of translational signaling in younger and older skeletal muscle.

The activation of the MPS machinery is driven to a great extent by the rapid postprandial increase in plasma leucine [3,4,10]. The provision of all EAAs are needed to support this effect, particularly following RE [60,61]. The postprandial leucinemia we observed in EAA and COM, irrespective of age, is consistent with that seen following whey protein ingestion in younger [4] and older adults [62]. It is recognized that higher daily and per-meal protein intakes are warranted in older adults [63], but not at the expense of energy intake [43,44]. Despite comparable aminoacidemia to whey with respect to leucine and the BCAAs, our gel formulation is non-satiating and does not compromise subsequent energy intake in older women [45,46]. The provision of large amounts of leucine may increase BCAA oxidation rates, limiting their availability to synthesise new protein; additionally, decreased isoleucine availability may also reduce intracellular leucine transport [50,64,65,66]. Hence, the formulation used in this study maintained the contribution from BCAAs in the presence of enriched leucine, and rather reduced the percentage of the remaining EAAs [50]. Recent work has demonstrated that high doses (6.4 g) of EAAs stimulate greater increases in whole-body protein synthesis and enhance suppression of whole-body protein breakdown compared to low EAA doses (3.2 g) and established whey protein formulations [67]. Thus, our formulation may possess additive value in augmenting suboptimal protein doses [68] or enhancing the anabolic effect of food as an adjunct to traditional protein sources [67]. 

The phosphorylation of Akt, an upstream regulator of mTORC1 [69], did not differ in pooled data, but exhibited a significant age x condition interaction in age group analyses. There was no appreciable change in Akt phosphorylation in our younger group across all trials; however, Akt phosphorylation was elevated in the older group compared with the younger group at 2 h following COM. A previous study using the same exercise stimulus, albeit bilateral as opposed to unilateral, found the strongest phosphorylation of Akt^Ser473^ 3 h post-exercise, and this did not differ by age [19]. A similar study from the same group found greater Akt^Ser473^ in the younger group 3 h following RE and 2 h after the ingestion of 20 g of EAA [24]. Plasma leucine concentrations increased to a similar extent in both groups up to 2 h following exercise [24], consistent with the findings of the present study. The absence of Akt phosphorylation following EAA ingestion was coupled with a stronger insulinemic response compared with COM. It is possible that the acute contractile stimuli in COM may have made the muscle more insulin-sensitive [70,71], thus augmenting Akt phosphorylation, and might partially explain the divergent findings from the young in the present study. 

Anabolic signaling targets within the mTORC1 pathway responsible for mRNA translation were robustly activated in both of our age groups. The phosphorylation of mTOR was amplified above baseline following COM in both groups, but to a greater extent in the young. The activation of S6K1 was significantly greater in the older group but post-hoc testing was unable to reveal the location of these differences. Given the large effects, it appears that EAA and COM may have induced greater S6K1 phosphorylation in the older group. These findings concur with those of Drummond et al. [24], who proposed the existence of delayed skeletal muscle anabolism in older adults. However, due to taking only a single post-ingestion biopsy, we would have been limited in our ability to detect the earlier phosphorylation of S6K1 in the young [25]. The phosphorylation of 4E-BP1 was increased in response to each variation of anabolic stimuli in the younger group, with the strongest effect in COM compared with RE and EAA alone. However, there was no significant deviation from baseline in the older group and no significant effects when data were pooled. In a similar cohort of older adults, McKendry et al. [56] found no increase in 4E-BP1^Thr37/46^ 1 h following a bout of acute RE. The same group also found no significant increase or age-related differences in 4E-BP1^Thr37/46^ 240 min after consuming 15 g of milk protein isolate [72]. In the present study, a subsequent biopsy at 3 h would have been informative, perhaps indicating a delay/resistance in 4E-BP1 signaling [24], or comparable post-exercise deactivation with their younger counterparts [19]. Interestingly, the phosphorylation of rpS6, a downstream component of mTORC1 signaling and a reliable surrogate of S6K1 activity [73], was robustly stimulated in both pooled and age group data, with an augmented effect in COM. These findings concur with those of Farnfield et al. [20], who observed robust increases in rpS6 phosphorylation after acute RE and whey protein ingestion in younger and older untrained adults. Collectively, these data underline the importance of combination strategies (i.e., dietary protein and RE) for stimulating this downstream regulator of cap-dependent translation. The age-related discrepancies in Akt, S6K1, and 4E-BP1 phosphorylation may be due to the delayed anabolic signaling response reported in older adults [24]. Previous work has shown differential activation of these signaling targets 30 min after RE and the ingestion of 30 g whey protein hydrolysate in younger and older adults [25]. We propose that any apparent effects in the younger group had normalized by the 2 h timepoint. Additional biopsies at 1, 3, 6, and 24 h would have provided further temporal insights on the regulation of anabolic signaling in this regard, given that MPS can be elevated for up to 24–72 h [24,25,56,74,75,76].

The phosphorylation and activation of FAK, a translator of cytoskeletal stress and strain signals via integrins [77], did not significantly differ by age or condition in pooled or age-group analyses. Nevertheless, a large between-group difference was found after RE (*d* = 0.82). The paucity of data in older humans on FAK limits our ability to speculate on these findings. In rodent models and young human males, overload induces FAK phosphorylation at tyrosine residues [78,79]. It is possible that increased FAK phosphorylation occurred in closer temporal proximity to the RE stress [80], or the acute mechanical stress of the RE was not sufficient to robustly increase FAK phosphorylation in either group [79,80]. Further exploration is warranted to confirm or deny the existence of age- and trial-related differences in FAK activation.

The basal protein content of the amino acid sensor Sestrin2 did not significantly differ between groups and is consistent with previous findings [81]. Acute RE, but not protein ingestion, appears to hyperphosphorylate Sestrin2 [82,83], strengthening its association with GATOR2 and inhibiting mTORC1 activation [84]. Previous studies have quantified Sestrin2 phosphorylation using the electrophoretic mobility shift method [81,82,83,84]. Had we implemented this, we may have gained further insights given the purported effects of leucine on the Sestrin2-GATOR2 interaction [85]. Moreover, recent studies have noted decreased protein content of other Sestrin isoforms (i.e., Sestrin1 and Sestrin3) in the resting skeletal muscle of older adults compared with the young [81]. Since the collection of our data, Sestrin1 has been implicated as a key mediator in the activation of mTORC1 by oral leucine, through its dissociation from GATOR2, and it may well possess the highest affinity for leucine among the Sestrins [86]. The emergence of Sestrins per se as nodal regulators of mammalian skeletal muscle growth [16] is an exciting area of further inquiry within the context of protein nutrition, exercise, aging, and health in humans.

Our findings must be interpreted with caution given the small sample size and large within-group variability. Notwithstanding, the sample size of both groups is indicative of similar literature in the area [22,24,27] and these early results provide a rationale for subsequent exploration. This study has similar limitations to those of Francaux et al. [25], in that we obtained only two biopsies per condition (i.e., one at baseline and one 2 h post-condition). Future studies should look to establish the efficacy of the protein gel formulation when consumed as part of a daily routine in older people. Previous work from our laboratory has demonstrated that the same amino acid formulation can enhance functional performance and body composition in older persons in the absence of exercise [50]. Hence, future work should investigate the more palatable version of our formulation [45,46] using novel, ecologically-valid measures of MPS that account for habitual living conditions outside of the laboratory environment, such as D_2_O tracer techniques [21,56,87,88]. The impact of the supplements on those of a more advanced age (i.e., Franzon et al. [89]), with lower whole-body strength and activity levels and/or polypharmacy [90] may be more representative of the older population and provide useful insights. It is important to note that none of our participants were characterized as sarcopenic using any established algorithm [31,91,92], yet five (*n* = 5) were below the cut-points for upper-body MQ [47].

## 5. Conclusions

These findings provide proof-of-concept that ingestion of a novel, gel-based, leucine-enriched EAA supplement results in substantial aminoacidemia and anabolic signaling in younger and older individuals. This study, in combination with our previous work, suggests that this formulation can augment dietary protein consumption, intracellular anabolic signaling, and aminoacidemia in older adults without deleterious effects on appetite and subsequent energy intake. Future research should explore the efficacy of these supplements in larger studies, using gold-standard techniques to evaluate MPS in habitual living conditions over the long-term. 

## Figures and Tables

**Table 1:** Baseline anthropometrics, body composition, functional performance, and strength characteristics of younger and older participants (mean ± SD).

	Younger (*n* = 7)	Older (*n* = 7)	Total (*n* = 14)	*p*-Value
**Anthropometrics**				
Age (years)	28 ± 4	71 ± 3	50 ± 23	<0.001
Height (cm)	175.3 ± 9.8	168.9 ± 11.8	172.1 ± 10.9	0.29
Body mass (kg)	83.2 ± 13.1	81.8 ± 17.3	82.5 ± 14.8	0.86
BMI (kg/m^2^)	27.0 ± 3.3	28.5 ± 4.0	27.8 ± 3.6	0.46
**Body Composition**				
Fat mass (kg)	22.1 ± 7.2	28.2 ± 8.2	25.2 ± 8.1	0.16
Lean mass (kg)	57.7 ± 10.7	49.6 ± 9.9	53.6 ± 10.7	0.17
%TFM	27.7 ± 7.8	35.8 ± 7.3	31.7 ± 8.4	0.07
ALM (kg)	27.5 ± 5.8	22.5 ± 5.8	25.0 ± 6.1	0.13
SMI (kg/m^2^)	8.9 ± 1.3	7.8 ± 1.1	8.3 ± 1.3	0.12
**Functional Performance**				
Gait speed (m/s)	1.3 ± 0.2	1.2 ± 0.3	1.3 ± 0.3	0.35
Dominant HGS (kg)	40.0 ± 11.8	30.2 ± 11.0	35.1 ± 12.1	0.13
Upper body MQ (kg/kg)	5.7 ± 1.0	5.2 ± 0.9	5.5 ± 0.9	0.40
Dominant leg 1RM (kg)	65.3 ± 21.5	30.2 ± 10.0	47.7 ± 24.3	0.004
Lower body MQ (kg/kg)	3.2 ± 0.6	1.8 ± 0.5	2.5 ± 0.9	<0.001
HOMA-IR *	2.3 ± 0.1	2.0 ± 0.6	2.1 ± 1.0	0.64

Abbreviations: %TFM, percentage tissue fat mass; 1RM, one-repetition maximum; ALM, appendicular lean mass; BMI, body mass index; HGS, handgrip strength; HOMA-IR, Homeostatic Model Assessment of Insulin Resistance; MQ, muscle quality; SMI, skeletal muscle index. * *n* = 5 per age group. *P* values relate to between-group comparisons.

**Table 2:** Essential amino acid composition of the gels (50 mL each) used in the EAA and COM trials.

Essential Amino Acid	g/gel	g/trial
Leucine	3.0	6.0
Isoleucine	0.8	1.6
Valine	0.9	1.8
Lysine	0.9	1.8
Histidine	0.4	0.8
Methionine	0.2	0.4
Phenylalanine	0.5	1.0
Threonine	0.8	1.6
Tryptophan	0.0	0.0
ΣBCAAs	4.7	9.4
ΣEAAs	7.5	15.0

Abbreviations: BCAA, branched-chain amino acid; EAA, essential amino acid.

**Table 3:** Plasma leucine, branched-chain amino acid, insulin, and glucose concentrations in younger and older adults for each condition.

Variable	Younger (*n* = 5)			Older (*n* = 5)		
	EAA	RE	COM	EAA	RE	COM
***Leucine***						
C_max_ (µM)	454 ± 32 ^a^	169 ± 27	537 ± 111 ^a^	418 ± 99	165 ± 37	553 ± 136 ^a^
T_max_ (min)	66 ± 6	42 ± 26	102 ± 12	90 ± 13	114 ± 6	78 ± 12 ^a^
AUC (µM·2 h)	41241 ± 2917 ^a^	17357 ± 2663	41600 ± 9171	37303 ± 10766	14687 ± 2707	44588 ± 10561
***ΣBCAAs***						
C_max_ (µM)	901 ± 92	519 ± 54	994 ± 266	878 ± 75 ^a^	414 ± 36	913 ± 141
T_max_ (min)	66 ± 6	36 ± 15	108 ± 12	72 ± 15	66 ± 17	72 ± 12
AUC (µM·2 h)	87492 ± 10073 ^a^	39589 ± 3919	71546 ± 12908	80553 ± 10146 ^a^	39716 ± 4123	85148 ± 10693
***Insulin***						
Baseline (µIU/mL)	9.7 ± 0.7	5.6 ± 0.7	6.9 ± 0.9	8.5 ± 2.0	10.0 ± 1.1	6.4 ± 1.8
C_max_ (µIU/mL)	20.2 ± 2.8 ^#^	11.1 ± 0.7 ^#^	15.8 ± 0.7 ^#^	28.3 ± 1.6 ^a,b,#^	18.2 ± 2.3 ^#^	21.3 ± 1.5 ^a,#^
T_max_ (min)	18 ± 12	48 ± 20	12 ± 7	30 ± 16	30 ± 23	6 ± 6
AUC (µIU/mL·2 h)	1508.4 ± 104.0 ^#^	999.6 ± 67.2	1416.3 ± 86.3	2347.2 ± 138.1 ^a,b,#^	1377.3 ± 238.3	1800.3 ± 208.6 ^a^
***Glucose***						
Baseline (mmol/L)	4.9 ± 0.1	5.3 ± 0.2	5.0 ± 0.2	5.1 ± 0.2	5.5 ± 0.3	5.3 ± 0.3
C_max_ (mmol/L)	5.5 ± 0.3	5.7 ± 0.3	5.8 ± 0.2	5.9 ± 0.5	5.9 ± 0.6	6.1 ± 0.4
T_max_ (min)	54 ± 22	18 ± 29	30 ± 19	18 ± 18	60 ± 19	24 ± 24
AUC (mmol/L·2 h)	624.3 ± 19.2	617.1 ± 12.0	631.2 ± 15.0	650.1 ± 27.5	637.5 ± 46.5	612.3 ± 38.6

Data are means ± SEM. ^a^ Significantly different from RE within the same age group; ^b^ significantly different from COM within the same age group; # denotes statistical significance between age groups within the same condition. Abbreviations*:* AUC, area under the curve; BCAA, branched-chain amino acids; C_max_, maximal concentration; COM, essential amino acids following resistance exercise; EAA, essential amino acids; RE, resistance exercise; T_max_, time to reach maximal concentration.
